# Cardiovascular Safety of Clonidine and Dexmedetomidine in Critically Ill Patients after Cardiac Surgery

**DOI:** 10.1155/2020/4750615

**Published:** 2020-05-07

**Authors:** Angelina Grest, Judith Kurmann, Markus Müller, Victor Jeger, Bernard Krüger, Donat R. Spahn, Dominique Bettex, Alain Rudiger

**Affiliations:** Institute for Anaesthesiology, University Zurich and University Hospital Zurich, Zürich, Switzerland

## Abstract

**Purpose:**

The aim of this retrospective study was to assess the haemodynamic adverse effects of clonidine and dexmedetomidine in critically ill patients after cardiac surgery.

**Methods:**

2769 patients were screened during the 30-month study period. Heart rate (HR), mean arterial pressure (MAP), and norepinephrine requirements were assessed 3-hourly during the first 12 hours of the continuous drug infusion. Results are given as median (interquartile range) or numbers (percentages).

**Results:**

Patients receiving clonidine (*n* = 193) were younger (66 (57–73) vs 70 (63–77) years, *p*=0.003) and had a lower SAPS II (35 (27–48) vs 41 (31–54), *p*=0.008) compared with patients receiving dexmedetomidine (*n* = 141). At the start of the drug infusion, HR (90 (75–100) vs 90 (80–105) bpm, *p*=0.028), MAP (70 (65–80) vs 70 (65–75) mmHg, *p*=0.093), and norepinephrine (0.05 (0.00–0.11) vs 0.12 (0.03–0.19) mcg/kg/min, *p* < 0.001) were recorded in patients with clonidine and dexmedetomidine. Bradycardia (HR < 60 bpm) developed in 7.8% with clonidine and 5.7% with dexmedetomidine (*p*=0.51). Between baseline and 12 hours, norepinephrine remained stable in the clonidine group (0.00 (−0.04–0.02) mcg/kg/min) and decreased in the dexmedetomidine group (−0.03 (−0.10–0.02) mcg/kg/min, *p*=0.007).

**Conclusions:**

Dexmedetomidine and the low-cost drug clonidine can both be used safely in selected patients after cardiac surgery.

## 1. Introduction

Maintaining an optimal level of comfort and safety for critically ill patients is a universal goal in postoperative care [1, 2]. This can be achieved with the use of alpha-2 adrenergic agents, in particular, clonidine and dexmedetomidine, which induce dose-dependent sedation, analgesia, and anxiolysis [3, 4]. Clonidine has traditionally been used in our institution for sedation in the perioperative setting and in critically ill patients after cardiac surgery [5–9]. Particular benefits of this sedative are its low acquisition costs. Adverse effects of clonidine are hypotension and bradycardia [10], which might limit its use in haemodynamic unstable patients.

In 2013, dexmedetomidine was introduced into the Swiss market. The number of reports describing the benefits of dexmedetomidine is growing continuously since then: dexmedetomidine reduced the lengths of mechanical ventilation and hospital stay, and it lowered the overall costs compared with that of propofol [11–13]. The perioperative use of dexmedetomidine was associated with a decreased incidence of postoperative complications, delirium, and mortality up to one year after cardiac surgery [14]. However, dexmedetomidine treatment is expensive and might not be universally available.

To date, there is a shortage of studies comparing dexmedetomidine and clonidine in the intensive care unit (ICU) setting. The only direct comparison between the two alpha-2 agonists available is limited by the low number of included patients (*n* = 35 per group) [15].

## 2. Materials and Methods

### 2.1. Objectives

The aim of this study was to assess the haemodynamic adverse effects of the alpha-2 agonists clonidine and dexmedetomidine in critically ill patients after cardiac surgery.

### 2.2. Design

The study is a retrospective observational study. The study protocol was approved by the ethics council of the Canton of Zurich, Switzerland (BASEC-Nr. PB_2016-00333). Patients' general consent was required for the screening of their charts and the retrospective analysis of their data.

### 2.3. Setting

The study was performed in the cardiosurgical ICU at the University Hospital Zurich, Switzerland. The treatment principles of patients hospitalized in this particular ICU have been summarized [16, 17]. In our practice, patients generally arrive from the operation room under propofol sedation. Alpha-2 agonists are second-line agents when prolonged sedation becomes necessary. In our clinical practice, clonidine is used in haemodynamically stable patients with norepinephrine requirements <0.1 mcg/kg/min, while dexmedetomidine is used in patients with a norepinephrine requirement <0.3 mcg/kg/min. The patients requiring higher norepinephrine support are usually sedated with midazolam. However, no strict sedation guidelines were applied during the study period, and the choice of sedative was at the discretion of the ICU consultant on call.

### 2.4. Data Collection

Demographic and baseline characteristics including the Simplified Acute Physiology Score (SAPS) II were collected for both groups. Higher SAPS values indicate more severe illness and a higher predicted mortality [18]. Medical information and laboratory data were extracted from the electronic patient information system. Medication and haemodynamic variables were collected from the handwritten patients' charts.

### 2.5. Population

All patients admitted to our cardiovascular ICU during an 18-month-period were screened for treatment with either clonidine (Catapresan®) and/or dexmedetomidine (Dexdor®). Study inclusion criteria for patients were treatment with either clonidine or dexmedetomidine. The exclusion criteria were absence of consent for screening, treatment with both clonidine and dexmedetomidine during the same ICU stay, only drug bolus administration in absence of a continuous drug infusion, absence of cardiac surgery, use of either extracorporeal life support, or ventricular assist devices. Patients were included in the analysis only once. Those receiving both clonidine and dexmedetomidine during the same ICU stay were not considered for this comparison.

### 2.6. Variables

Heart rate, mean arterial pressure, and norepinephrine requirements were recorded 3-hourly. The observation period started with the beginning of the alpha-2 agonist infusion and ended 12-hours later. Bradycardia was defined as a decrease of the heart rate below 60 beats per minute.

### 2.7. Study Size

The study size was defined by the 30-month screening period (convenience sample).

### 2.8. Statistics

Median (interquartile range) and numbers (percentages) were calculated for the overall sample and subgroups. Missing values were replaced by the last recorded value (carrying forward). Comparisons were made with the use of the Mann–Whitney *U* test, Fisher's exact test, or the chi-squared test, as appropriate. The null hypothesis was rejected with a two-sided *p* value < 0.05. All analyses were performed with the use of SPSS 24 for Windows 10.

## 3. Results

**Figure 1:**
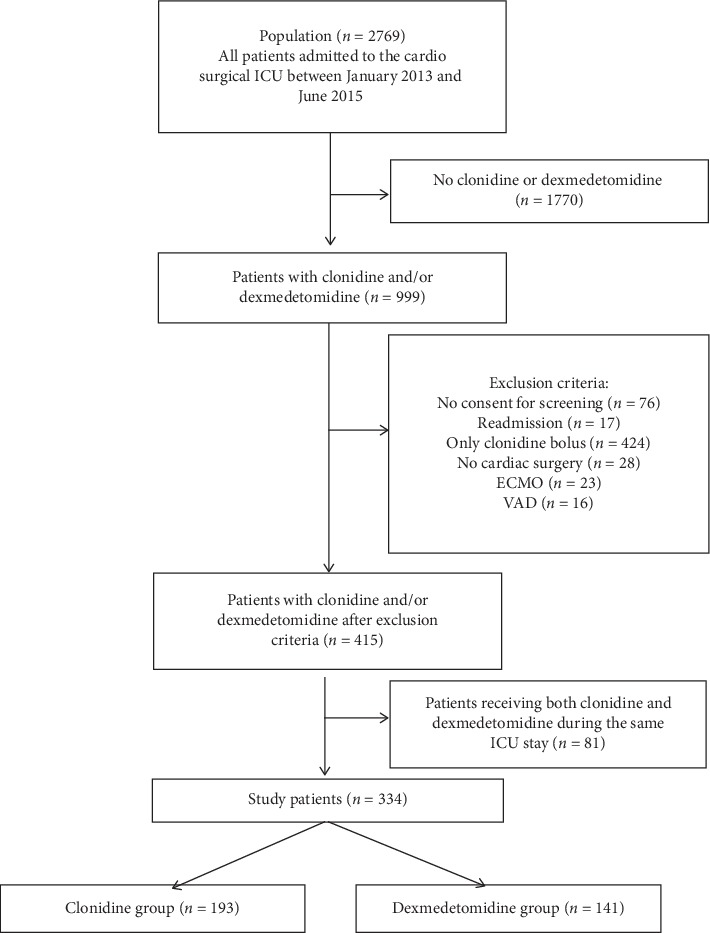
Study population with inclusion and exclusion criteria. ECMO: extracorporeal membrane oxygenation; VAD: ventricular assist device; ICU: intensive care unit.

During the 30-month study period, 2769 patients were admitted to the cardiosurgical ICU. A summary of included and excluded patients is given in Figure 1. Eventually, this study compared 193 patients sedated with clonidine and 141 patients sedated with dexmedetomidine. Patient characteristics are displayed in Table 1.

All patients underwent cardiac surgery, which included valve surgery in 179 (54%), coronary artery bypass surgery in 137 (41%), aortic dissection surgery in 35 (10.5%), aortic aneurysm surgery in 50 (15%), other major vascular surgery in 25 (7.5%), myocardial resection in 5 (1.5%), implantable cardioverter defibrillator implantation in 12 (3.6%), and heart transplantation in 9 (2.7%) patients. Several patients received combinations of the above-listed interventions. Cardiopulmonary bypass was required in 251 (75%) of the operations without significant difference between the two patient groups (*p*=0.31). The postoperative left ventricular ejection fraction in both groups was 55 (45–60)%; *p*=0.16.

**Figure 2:**
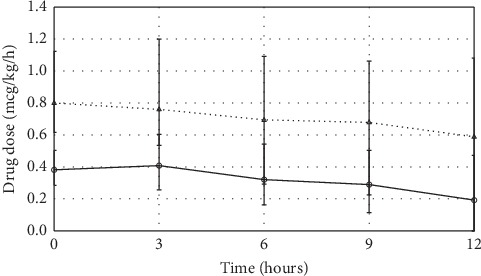
Drug dosages during dexmedetomidine and clonidine infusion. The clonidine and dexmedetomidine doses decreased by −0.14 (−0.35–0.00) mcg/kg/h and −0.12 (−0.69–0.21), respectively. Values indicate median ± interquartile range. Solid line (──) indicates clonidine patients; dashed line (- - -) indicates dexmedetomidine patients. Values represent median (interquartile range).

The average initial dose of clonidine was 0.38 (0.29–0.50) mcg/kg/h. The average initial dexmedetomidine dose was 0.80 (0.61–1.12) mcg/kg/h. Both drugs were started on ICU day 2 (1–3); *p*=0.18. Patients were treated with clonidine for a total duration of 16 (11–50) hours. In the dexmedetomidine group, patients received the drug infusion for a total duration of 31 (13–68) hours. Study drug doses over time are displayed in Figure 2.

**Figure 3:**
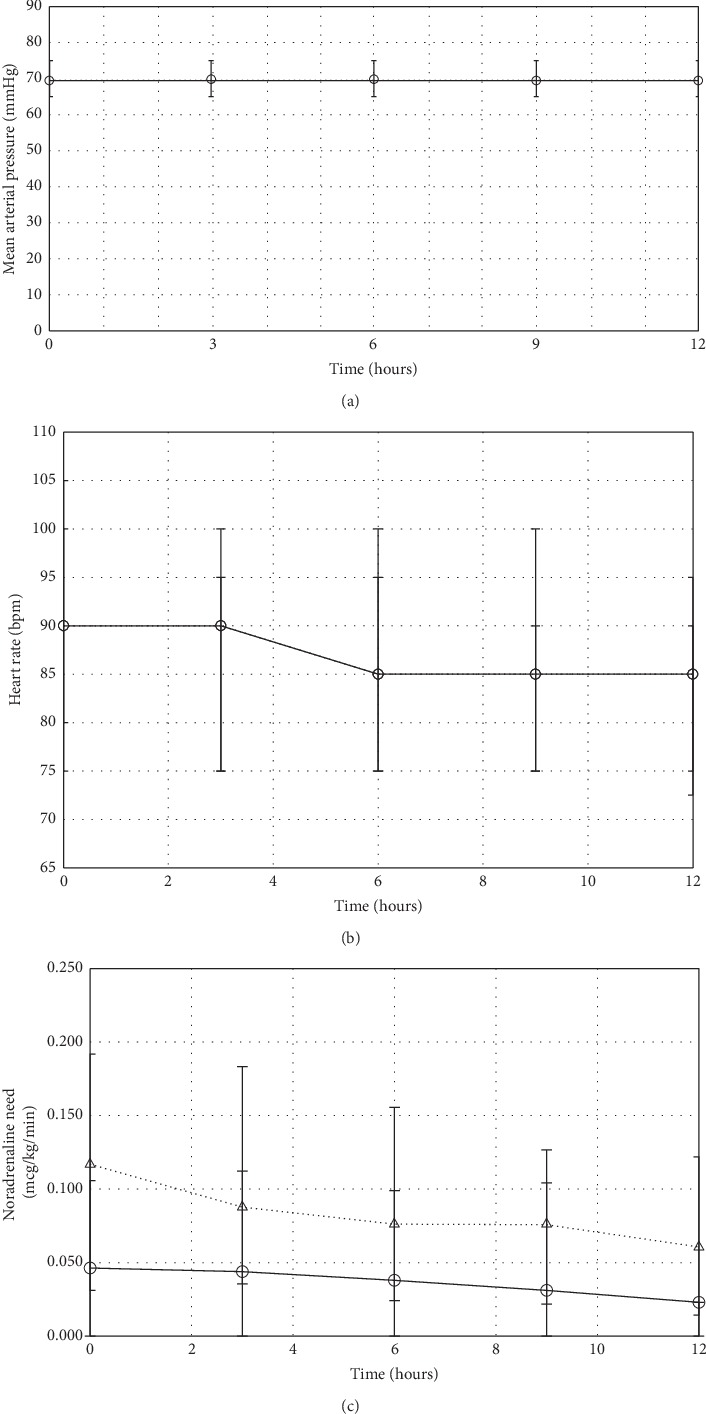
Hemodynamic variables during dexmedetomidine and clonidine infusion. Solid line (──) indicates clonidine patients; dashed line (- - -) indicates dexmedetomidine patients. Values represent median (interquartile range).

Haemodynamic variables, laboratory values, and ICU management at the start of clonidine or dexmedetomidine treatment are shown in Table 2. As shown in Figure 3(a), the median arterial pressure remained stable in both groups during the twelve-hour observation period. Heart rate decreased in both groups by −10 (−20–0) bpm (*p*=0.33), Figure 3(b). Bradycardia developed in 7.8% of the patients treated with clonidine and 5.7% of patients treated with dexmedetomidine (*p*=0.52). At the beginning of the alpa-2 agonist infusion, norepinephrine requirements were higher in patients receiving dexmedetomidine (0.05 (0.00–0.11) vs 0.12 (0.03–0.19) mcg/kg/min, *p* < 0.001). Between baseline and the twelve-hour follow-up, the norepinephrine requirements remained stable in the clonidine group (0.00 (−0.04–0.02) mcg/kg/min), whereas a decrease of norepinephrine was seen in the dexmedetomidine group (−0.03 (−0.10–0.02) mcg/kg/min, *p*=0.007) (Figure 3(c). During the 12-hour observation period, a transient increase in norepinephrine was recorded in 95 (49%) patients treated with clonidine and 60 (43%) patients treated with dexmedetomidine (*p*=0.27) by 0.04 (0.03–0.08) and 0.06 (0.03–0.12) mcg/kg/min (*p*=0.014), respectively.

## 4. Discussion

This retrospective analysis demonstrates that both clonidine and dexmedetomidine can be used safely for sedation of patients after cardiac surgery hospitalized in the ICU.

### 4.1. Population

Patients receiving dexmedetomidine were older and had a higher SAPS II score and a higher requirement for norepinephrine at the start of the drug infusion, indicating that these patients had a higher severity of illness than the patients treated with clonidine. This reflects our clinical practice that clonidine is used in haemodynamically more stable patients. This translated in a longer ICU length of stay in the dexmedetomidine group, while mortality was low in both groups.

### 4.2. Clonidine

We used clonidine in a median dose of 0.4 mcg/kg/h, while Srivastava et al. used a mean dosage of 1.4 mcg/kg/h. Haemodynamic instability is one of the most frequently mentioned side-effects of clonidine [10]. Srivastava et al. described the frequencies for bradycardia (heart rate below 50 bpm) and hypotension (systolic blood pressure below 80 mmHg or diastolic blood pressure below 50 mmHg) during clonidine infusion with 8.6% (3/35) and 31% (11/35), respectively [15]. In our ICU setting, the overall risk of bradycardia was below ten percent and mean arterial blood pressure remained stable due to adjustments of the norepinephrine doses. Overall, norepinephrine requirements did not increase during the 12-hour observation period.

The major advantages of clonidine are its low costs. A 24-hour treatment of an average study patient (body weight 79 kg, clonidine dose 0.4 mcg/kg/h) costs about 18 Euros. Based on our findings, we can recommend clonidine for sedation in ICU patients after cardiac surgery with norepinephrine requirements up to 0.1 mcg/kg/min at the beginning of the clonidine infusion, provided the patients are adequately monitored and managed.

### 4.3. Dexmedetomidine

In the dexmedetomidine group, patients were treated with a median dose of 0.8 mcg/kg/h, while Srivastava et al. used a mean dose of only 0.4 mcg/kg/h. In the Midex and Prodex studies, the median dose of dexmedetomidine was 0.45 and 0.96 mcg/kg/h, respectively [19]. Hypotension (no definition provided) occurred in 21% (51/247) in the Midex study and in 13% (32/246) in the Prodex study [19]. Srivastava et al. described the frequencies for bradycardia (heart rate below 50 bpm) and hypotension (systolic blood pressure below 80 mmHg or diastolic blood pressure below 50 mmHg) during dexmedetomidine infusion with 11% (4/35) and 8.6% (3/35), respectively [15]. In our study, mean arterial blood pressure in the dexmedetomidine group remained stable over time. Less than half of the patients being treated with dexmedetomidine showed a transient increase of norepinephrine, while the overall norepinephrine need was decreasing during the observation period. This suggests that dexmedetomidine provides good haemodynamic stability.

In the Midex and Prodex studies, the risk of bradycardia (no definition provided) was 14.2% (35/247) and 13.0% (32/246), respectively [19]. In our study, the incidence of bradycardia was 5.7% in the dexmedetomidine group.

Up to now, merely one existing study directly compared the two alpha-2 agonists in the ICU setting. Srivastava's findings concerning the haemodynamic stability conform to our findings, namely, that dexmedetomidine showed a better haemodynamic stability than clonidine. However, due to distinctive drug dosages, it is not possible to make a proper comparison between the two studies: While we used twice as much dexmedetomidine, our clonidine infusion doses were lower by a factor of three. Additionally, Srivastava's study is limited by the small study group of 70 patients [15]. Recently, Morelli et al. investigated the effect of dexmedetomidine sedation on norepinephrine requirements in 38 septic shock patients in a crossover trial. Four hours after the stop of propofol and the initiation of a dexmedetomidine infusion of 0.7 ± 0.2 mcg/kg/h, the norepinephrine dose decreased from 0.69 ± 0.72 mcg/kg/min to 0.30 ± 0.25 *μ*·g/kg/min (*p* < 0.005). Back on propofol 8 hours after stopping dexmedetomidine, norepinephrine increased again to 0.42 ± 0.36 *μ*g/kg/min (*p* < 0.005) [20]. While the study population was different and the norepinephrine doses at baseline were much higher, the dexmedetomidine doses administered and the trend of the norepinephrine changes were similar, supporting in part the findings of the present study.

The costs for a 24-hour treatment of dexmedetomidine for an average study patient (body weight 78 kg, dexmedetomidine dose 0.8 mcg/kg/h) are approximately 224 Euros. Hence, dexmedetomidine therapy is exceeding the costs of clonidine by more than ten times. While clonidine is a good choice for haemodynamically stable patients (NA ≤ 0.1 mcg/kg/min), our study results suggest that dexmedetomidine is a safe option for patients with a moderate haemodynamic instability (norepinephrine > 0.1, but ≤ 0.2 mcg/kg/min). For highly unstable patients (norepinephrine > 0.2 mcg/kg/min), a treatment with another sedative (e.g., midazolam) may be considered.

Based on these data, prospective studies can be planned for a direct comparison between the two alpha-2 agonists, potentially also in patients with haemodynamic instability. More data regarding antidelirogenic effects and impact on length of mechanical ventilation or length of stay are needed for clonidine or dexmedetomidine treatments. As treatment with clonidine is inexpensive compared with dexmedetomidine, further research would be of economic interest for hospital administrators and public health.

### 4.4. Limitations

Our study has some important limitations. First, this study has a retrospective design, and the patients were not randomly allocated to a patient group. As a result, the two patient groups have different baseline characteristics, making a direct head-to-head comparison of the two drugs impossible. However, this study represents a real-life experience giving information on clinical practice in our institution.

Second, all study variables were assessed 3-hourly and reflect more the overall trend than short-term fluctuations. Hence, if the patients showed relevant changes between the assessment times, we might have missed those. Clearly, not every variable of the patient condition (e.g., altertness and pain) and ICU interventions (e.g., fluid administration, analgesics, and termination of propofol) that may affect blood pressure and heart rate was collected and analysed in this study. In addition, some patients in our ICU had transient epicardial pacemakers with the possibility of pacing for haemodynamic reasons. This may have had an impact on the incidence of bradycardia in our study. Nevertheless, our results reflect haemodynamic changes in critically ill patients after cardiac surgery treated with alpha-2 agonists under every day conditions.

## 5. Conclusions

In patients treated with clonidine or dexmedetomidine, the incidence of bradycardia was below 10% and not different between groups. With clonidine, half of the patients required a mild increase in norepinephrine, while the overall norepinephrine use remained constant over 12 hours. With dexmedetomidine, less than half of the patients required a norepinephrine increase, while the overall norepinephrine use decreased during the observation period. This suggests that both dexmedetomidine and the lower-cost drug clonidine can be used safely for sedation in selected patients after cardiac surgery.

## Figures and Tables

**Table 1:** Patient characteristics.

Variable	All (*n* = 334)	Clonidine group (*n* = 193)	Dexmedetomidine group (*n* = 141)	*p* value
Age (years)	68 (58–75)	66 (57–73)	70 (63–77)	**0.003**
Male gender	256 (77%)	145 (75%)	111 (79%)	0.513
Weight (kg)	78 (68–90)	79 (69–91)	78 (68–88)	0.188
SAPS II (points)	37 (29–50)	35 (27–48)	41 (31–54)	**0.008**
ICU length of stay (days)	5 (2–8)	4 (2–7)	6 (3–8)	**0.004**
ICU mortality	12 (3.6%)	7 (3.6%)	5 (3.5%)	1.000

ICU: intensive care unit; SAPS: simplified acute physiology score.

**Table 2:** Physiological, laboratory, and treatment variables at the start of clonidine or dexmedetomidine therapy.

Variable	All (*n* = 334)	Clonidine group (*n* = 193)	Dexmedetomidine group (*n* = 141)	*p* value
Hemodynamic parameters				
(i) Heart rate (1/min)	90 (80–100)	90 (75–100)	90 (80–105)	**0.028**
(ii) Mean arterial pressure (mmHg)	70 (65–75) *n* = 333	70 (65–80) *n* = 192	70 (65–75)	0.093
(iii) Central venous pressure (mmHg)	10 (7–12) *n* = 322	9 (6–12) *n* = 185	10 (8–13) *n* = 137	**0.025**
(iv) Cardiac index (l/m [2]/min)	2.7 (2.5–3.3) *n* = 51	2.6 (2.4–3.0) *n* = 19	2.8 (2.6–3.5) *n* = 32	0.101
(v) SvO_2_ (%)	69 (63–75) *n* = 239	69 (64–75) *n* = 173	65 (59–75) *n* = 66	0.060

Laboratory values				
(i) Haemoglobin (g/l)	91 (83–103) *n* = 331	94 (85–107)	88 (80–97) *n* = 138	**0.002**
(ii) White blood cell count (G/l)	11.5 (8.8–15.1) *n* = 331	11.8 (8.8–15.1) *n* = 191	11.1 (8.6–15.1) *n* = 140	0.498
(iii) C-reactive protein (mg/l)	62 (14–131) *n* = 326	53 (6–133) *n* = 185	69 (34–129)	0.100
(iv) Procalcitonin (mcg/l)	1.91 (0.52–6.25) *n* = 46	1.43 (0.31–4.10) *n* = 28	3.46 (1.10–17.07) *n* = 18	0.075
(v) Creatinine (mcmol/l)	99 (78–141)	94 (76–133)	110 (83–150)	**0.005**
(vi) Aspartate transaminase (U/l)	62 (39–119) *n* = 322	55 (35–107) *n* = 184	71 (44–133) *n* = 138	**0.026**
(vii) Alanine transaminase (U/l)	27 (17–58) *n* = 325	27 (16–58) *n* = 188	28 (17–63) *n* = 137	0.606
(viii) Creatine kinase (U/l)	474 (242–847) *n* = 329	484 (228–889) *n* = 188	468 (255–807) *n* = 141	0.919
(ix) Myoglobin (mcg/l)	371 (218–767) *n* = 325	378 (229–670) *n* = 186	368 (214–898) *n* = 139	0.640
(x) Troponin (mcg/l)	0.81 (0.35–1.69) *n* = 320	0.78 (0.30–1.54) *n* = 184	0.84 (0.43–1.97) *n* = 136	0.135
(xi) Arterial lactate (mmol/l)	1.5 (1.0–2.3) *n* = 330	1.4 (1.05–2.2)	1.5 (1.0–2.6) *n* = 137	0.650
(xii) Arterial base excess (mmol/l)	−1.1 (−3.0–0.3) *n* = 330	−1.2 (−2.7–0.3)	−1.0 (−3.3–0.4) *n* = 137	0.574

Treatments				
Propofol *n* (%)	127 (38%)	70 (36%)	57 (40%)	0.494

Mechanical ventilation				
(i) Invasive *n* (%)	235 (70%)	124 (64%)	111 (79%)	**0.017**
(ii) Noninvasive *n* (%)	7 (2.1 %)	5 (2.6%)	2 (1.4%)	
Renal replacement therapy-*n* (%)	34 (10.2 %)	23 (12%)	11 (7.8%)	0.235
Norepinephrine *n* (%)	248 (74 %)	133 (69 %)	115 (82%)	**0.011**

Inotropes				
(i) Milrinone *n* (%)	80 (24%)	35 (18%)	45 (32%)	**0.004**
(ii) Epinephrine *n* (%)	73 (22%)	33 (17%)	40 (28%)	**0.016**

SvO_2_: central venous oxygen saturation. Variables were collected at start of clonidine or dexmedetomidine treatment.

## Data Availability

The data used to support this study are available from the corresponding author upon request.

## Abbreviations

HR: Heart rate
ICU: Intensive care unit
MAP: Mean arterial pressure
SAPS: Simplified acute physiology score.

## Additional Points

To date, there is a shortage of studies comparing the cardiovascular safety of dexmedetomidine and clonidine in the intensive care unit setting. This retrospective study assesses the hemodynamic adverse effects of clonidine and dexmedetomidine in critically ill patients after cardiac surgery. Between baseline and 12 hours, norepinephrine requirements remained stable in the clonidine group and decreased in the dexmedetomidine group. Bradycardia (HR < 60 bpm) developed in less than 10% of patients with no difference between groups. Dexmedetomidine and the low-cost drug clonidine can both be used safely in selected patients after cardiac surgery.
